# Coronary artery disease diagnosis with signal processing and machine learning of heart sound signals: a systematic review

**DOI:** 10.1038/s41746-026-02530-8

**Published:** 2026-03-16

**Authors:** Aikeliyaer Ainiwaer, Tom J.A.J. Konings, Kaisaierjiang Kadier, Xiang Ma, Muhammet Emin Akpulat, Frits W. Prinzen, Tammo Delhaas, Hongxing Luo

**Affiliations:** 1 Department of Physiology, Cardiovascular Research Institute Maastricht (CARIM), Maastricht University, Maastricht, the Netherlands; 2 Department of Biomedical Engineering, Cardiovascular Research Institute Maastricht (CARIM), Maastricht University, Maastricht, the Netherlands; 3 Department of Cardiology, First Affiliated Hospital of Xinjiang Medical University, Urumqi, China; 4 Institute for Surgical Research, Oslo University Hospital, Rikshospitalet, Postboks 4950, Nydalen, 0424 Oslo, Norway

**Keywords:** Cardiology, Computational biology and bioinformatics, Diseases, Health care, Medical research

## Abstract

Coronary artery disease (CAD) remains a major contributor to morbidity and mortality worldwide. Heart sound analysis has been investigated as a noninvasive approach to CAD detection, although existing evidence has been inconsistent. This systematic review evaluated the diagnostic performance of heart sound analysis for identifying CAD (≥50% stenosis). A search of four databases identified 1082 records, among which 40 studies involving 13,814 participants met the inclusion criteria. Among the 21 studies using signal processing methods, all but one of the larger studies (>50 participants, *n* = 15) reported diagnostic accuracy below 75%. The majority of signal processing studies lacked validation on independent datasets, thereby limiting confidence in the reliability of their reported performance. In contrast, 15 of the 19 studies applying machine learning-based methods reported accuracy, sensitivity, and specificity consistently above 80%. Moreover, 15 of these 19 studies conducted independent dataset validation, indicating comparatively stronger generalizability. Studies that used the full heart sound signal as model input also tended to achieve higher sensitivity than those using only the diastolic component, suggesting that utilizing the complete waveform preserves diagnostically informative features. These findings indicate that machine learning-based heart sound analysis may have diagnostic value for CAD, and larger multicenter studies are needed to further assess its clinical applicability and robustness.

## Introduction

Cardiovascular diseases cause more than 17 million deaths annually, with coronary artery disease (CAD) among the principal contributors^1^. Coronary angiography remains the diagnostic gold standard^2^, but it is invasive, costly, and associated with risks, such as bleeding, infection, and vessel injury^3^. Noninvasive-imaging techniques, including computed tomography angiography and magnetic resonance angiography, can reduce procedural risk, though they are generally time-consuming^4^. Ideally, CAD could be detected rapidly and at the bedside^3,4^.

Acoustic detection has been increasingly investigated as a noninvasive tool for identifying coronary artery stenosis. Its rationale is grounded in pathophysiological changes in heart sound characteristics. Turbulent and high-velocity flow through a stenotic coronary artery may generate intracoronary murmurs and high-frequency vibrations during diastole, observable in phonocardiographic recordings^5–8^. In addition, stenosis can reduce coronary perfusion and myocardial oxygen delivery, which may weaken ventricular contractility and delay valve closure. These hemodynamic changes may subsequently reduce the amplitude of the first and second heart sounds (S1 and S2) and increase the prominence of the third and fourth heart sounds (S3 and S4)^9–11^.

Several studies have reported that specific acoustic features derived through signal processing methods, such as high-frequency diastolic energy, can be predictive of CAD, while others have reported less conclusive findings^12,13^. These inconsistencies may be related to the limited generalizability of conventional signal-processing approaches across different patient populations. More recent work has applied machine learning (ML) methods, including deep learning, which can automatically learn and integrate multidimensional acoustic representations^14,15^. These studies have generally reported higher diagnostic accuracy than physiology-based signal analysis. However, differences in patient characteristics, recording conditions, sensor hardware, analytic pipelines, and validation strategies contribute to variability across studies. A systematic review is therefore needed to provide a comprehensive and comparative evaluation of signal processing and ML approaches for acoustic CAD diagnosis.

This review aims to summarize current signal processing and ML methods for heart sound-based CAD diagnosis, evaluate the diagnostic performance of these approaches, and identify priorities for future data collection, signal analysis, and clinical translation.

## Results

### Identification of relevant literature

**Fig. 1:**
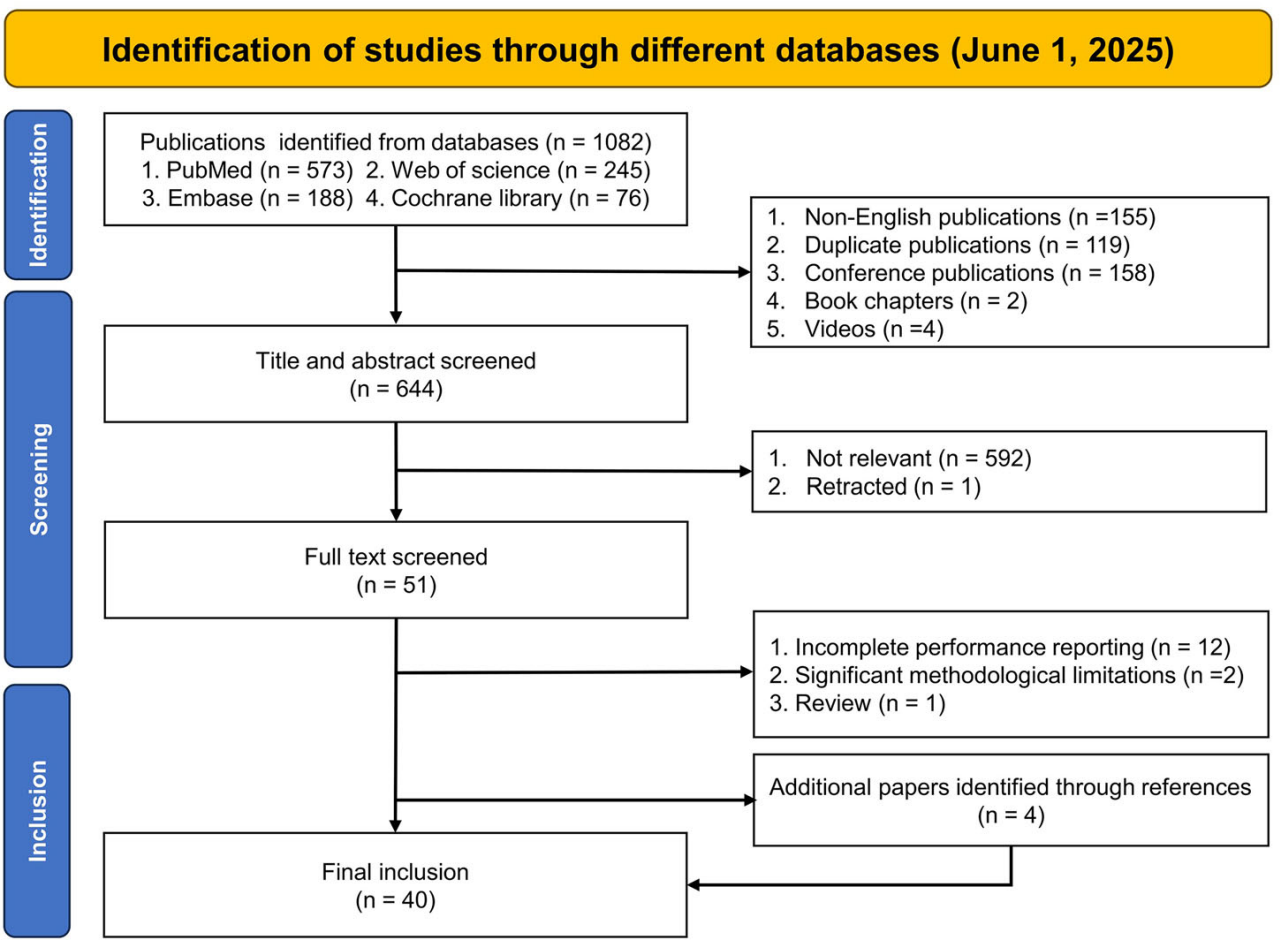
Preferred reporting items for systematic reviews and meta-analyses (PRISMA) diagram.

A total of 1082 publications were retrieved from PubMed (*n* = 573), Web of Science (*n* = 245), Embase (*n* = 188), and the Cochrane Library (*n* = 76) (Fig. 1). After excluding non-English publications (*n* = 155), duplicates (*n* = 119), conference papers (*n* = 158), book chapters (*n* = 2), and videos (*n* = 4), 644 records remained for title and abstract screening. Of these, 593 were excluded as not relevant to the research topic, and one was excluded due to retraction, leaving 51 articles for full-text review. Fifteen were then excluded because of insufficient reporting of diagnostic performance (*n* = 12), major methodological concerns (*n* = 2), or identification as a review article (*n* = 1). Screening the reference lists of remaining papers yielded four additional eligible studies. The final dataset, therefore, consisted of 40 studies including 13,814 participants. The median sample size was 128 (25th–75th percentile: 77–220), and publication years ranged from 1983 to 2025. Among these studies, 21 (53%) used signal processing approaches, and 19 (47%) used ML methods.

### Study overview

CAD was confirmed by coronary angiography in 38 of 40 eligible studies (Supplementary Table S1). Among these, 33 specified an explicit stenosis criterion: 32 defined CAD as at least 50% stenosis in any main coronary artery, whereas one study used a threshold >25%. The remaining seven studies distinguished “CAD” from “non-CAD” without mentioning a specific cutoff.

Table 1 summarizes the study characteristics, including filtering strategies, inclusion/exclusion of valvular disease, recording environments, breathing instructions, and microphone specifications. Although the frequency bands used for heart sound filtering varied, most studies analyzed signals within 20–1000 Hz. Since 2009, several studies have noted that coexisting cardiac murmurs may influence acoustic measurements. Echocardiography was therefore used in many cases to exclude primary valvular disease and to document CAD-associated secondary findings such as mitral regurgitation. Environmental noise control was reported in 26 studies, which recorded heart sounds in a quiet room, and 27 studies instructed participants to hold their breath during measurement.

**Table 1:** Signal processing (indicated in bold) and machine learning (indicated in italic) studies on heart sound-based CAD diagnosis^a^

Author (year)	Quiet room	Valvular disease excluded?	Breath holding	Filter range (Hz)	Microphones					
					Room mic	Mic attached to chest?	Channels	Bandwidth (Hz)	Sampling rate (Hz)	Resolution (bit)
**Semmlow**^12^ **(1983)**	**Yes**	**No**	**Yes**	**~400**	**No**	**Yes**	**1**	**20–600**	**1000**	
**Akay**^25^ **(1991)**	**Yes**	**No**	**Yes**	**200–1200**	**No**	**Yes**	**1**	**200–800**	**4000**	
*Akay*^33^ *(1991)*	*No*	*No*	*No*	*200–1200*	*No*	*No*	*1*	*200–800*	*4000*	
**Akay**^31^ **(1992)**	**No**	**No**	**Yes**	**350–1200**	**No**	**No**	**1**	**200–800**	**4000**	
**Akay**^16^ **(1992)**	**No**	**Yes**	**Yes**	**350–1200**	**No**	**No**	**1**	**200–800**	**4000**	
*Akay*^39^ *(1992)*	*Yes*	*No*	*Yes*	*200–1200*	*No*	*No*	*1*	*200–800*	*4000*	
**Akay**^20^ (1993)	Yes	No	Yes	180–1200	No	No	1	200–800	4000	
*Akay*^34^ *(1993)*	*Yes*	*No*	*Yes*	*180–1200*	*No*	*No*	*1*	*200–800*	*4000*	
**Akay**^21^ **(1993)**	**Yes**	**No**	**Yes**	**180–1200**	**No**	**No**	**1**	**200–800**	**4000**	
**Akay**^57^ **(1995)**	**No**	**No**	**Yes**	**180–1200**	**No**	**No**	**1**	**200–800**	**4000**	
**Tateishi**^42^ **(2001)**	**No**	**No**	**Yes**	**400–1500**	**No**	**Yes**	**5**		**24,000**	
*Zhao*^18^ *(2005)*	*No*	*No*	*Yes*		*No*	*No*	*1*		*2000*	
Akay^58^ (2009)	No	Yes	Yes	60–500	No	Yes	1	15–2000	4000	
*Griffel*^49^ *(2012)*	*Yes*	*Yes*	*Yes*		*No*	*Yes*	*4*		*22,099*	*16*
*Griffel*^59^ *(2013)*	*Yes*	*Yes*	*Yes*	*~2000*	*No*	*Yes*	*4*	*400–2700*	*22,099*	*16*
**Makaryus**^60^ **(2013)**	**No**	**Yes**	**No**		**Yes**	**Yes**	1*9	**400–2700**	**–**	
**Schmidt**^26^ **(2015)**	**No**	**Yes**	**No**	**25–250** **125–750** **250–1000**	**No**	**No**	**1**	**20–1000**	**4000**	**12**
**Azimpour**^61^ **(2016)**	**No**	**No**	**No**		**No**	**No**	**1**	**20–1000**	**4000**	**12**
**Winther**^29^ **(2016)**	**No**	**Yes**	**Yes**		**No**	**No**	**1**	**400–2700**	**8000**	**16**
**Winther**^28^ **(2017)**	**Yes**	**Yes**	**Yes**		**Yes**	**Yes**	**1**		**8000**	**16**
**Schmidt**^27^ **(2019)**	**Yes**	**Yes**	**Yes**		**Yes**	**Yes**	**1**			
*Samanta*^52^ *(2019)*	*Yes*	*No*	*No*	*20–1000*	*No*	*Yes*	*4*	*20–1000*	*8000*	*12*
*Li*^47^ *(2020)*	*Yes*	*Yes*	*No*	*20–1000*	*No*	*Yes*	*1*	*20–1000*	*1000*	
*Pathak*^62^ *(2020)*	*Yes*	*No*	*No*		*No*	*Yes*	*1*	*20–2000*	*44,100*	*16*
**Winther**^32^ **(2021)**	**Yes**	**Yes**	**Yes**	**20–1000**	**Yes**	**Yes**	**1**		**8000**	**16**
*Iqtidar*^40^ *(2021)*	*No*	*No*	*No*		*No*	*No*	*1*		*44,100*	*16*
*Liu*^15^ *(2021)*	*No*	*Yes*	*No*	*50–2000*	*No*	*Yes*	*5*		*2000*	
**Renker**^63^ **(2021)**	**Yes**	**Yes**	**Yes**		**Yes**	**Yes**	**1**			
**Larsen**^41^ **(2022)**	**Yes**	**Yes**	**Yes**	**~1000**	**Yes**	**Yes**	**1**		**8000**	**16**
*Pathak*^36^ *(2022)*	*Yes*	*No*	*Yes*	*~500*	*No*	*Yes*	*4*		*8000*	
*Khan*^64^ *(2022)*	*Yes*	*Yes*	*Yes*	*20–1200*	*No*	*Yes*	*4*		*8000*	*16*
*Huang*^38^ *(2022)*	*Yes*	*Yes*	*Yes*		*No*	*Yes*	*1*		*44,100*	*16*
**Schmidt**^65^ **(2022)**	**Yes**	**Yes**	**Yes**		**Yes**	**Yes**	**1**		**8000**	**16**
**Lehmacher**^13^ **(2022)**	**No**	**Yes**	**Yes**		**Yes**	**Yes**	**1**		**8000**	**16**
**Rasmussen**^30^ **(2023)**	**No**	**Yes**	**Yes**		**Yes**	**Yes**	**1**		**8000**	**16**
*Ainiwaer*^14^ *(2023)*	*Yes*	*Yes*	*No*	*50–500*	*No*	*No*	1*9^b^	*20–2000*	*8000*	*16*
*Dai*^37^ *(2024)*	*Yes*	*Yes*	*No*	*50–500*	*No*	*No*	1*9^b^	*20–2000*	*8000*	*16*
*Yin*^66^ *(2024)*	*Yes*	*No*	*No*		*No*	*Yes*	*1*		*8000*	
*Liu*^67^ *(2025)*	*Yes*	*Yes*	*No*	*25–400*	*No*	*Yes*	*1*	*20–1000*	*2000*	*16*
*Fynn*^68^ *(2025)*	*Yes*	*Yes*	*Yes*	*20–800*	*No*	*Yes*	*8*	*20–1000*	*2000*	*16*

*Hz* hertz, *Mic* microphone.

^a^Empty cells indicate that the data are not provided in the publications.

^b^In these studies, a microphone was used to collect heart sounds from 9 positions on the precordial area.

Because microphone characteristics directly affect signal quality, Table 1 also reports microphone parameters. Nine studies used an additional microphone to record ambient noise for later subtraction. In 25 studies (63%), the primary microphone was secured with straps to ensure stable placement, whereas the remaining studies used manual positioning. Most investigations used a single microphone with bandwidths between 20 and 2700 Hz, sampling rates between 1000 and 44,100 Hz, and bit depth of 12 or 16.

### Heart sound features for CAD diagnosis in signal processing studies

**Fig. 2:**
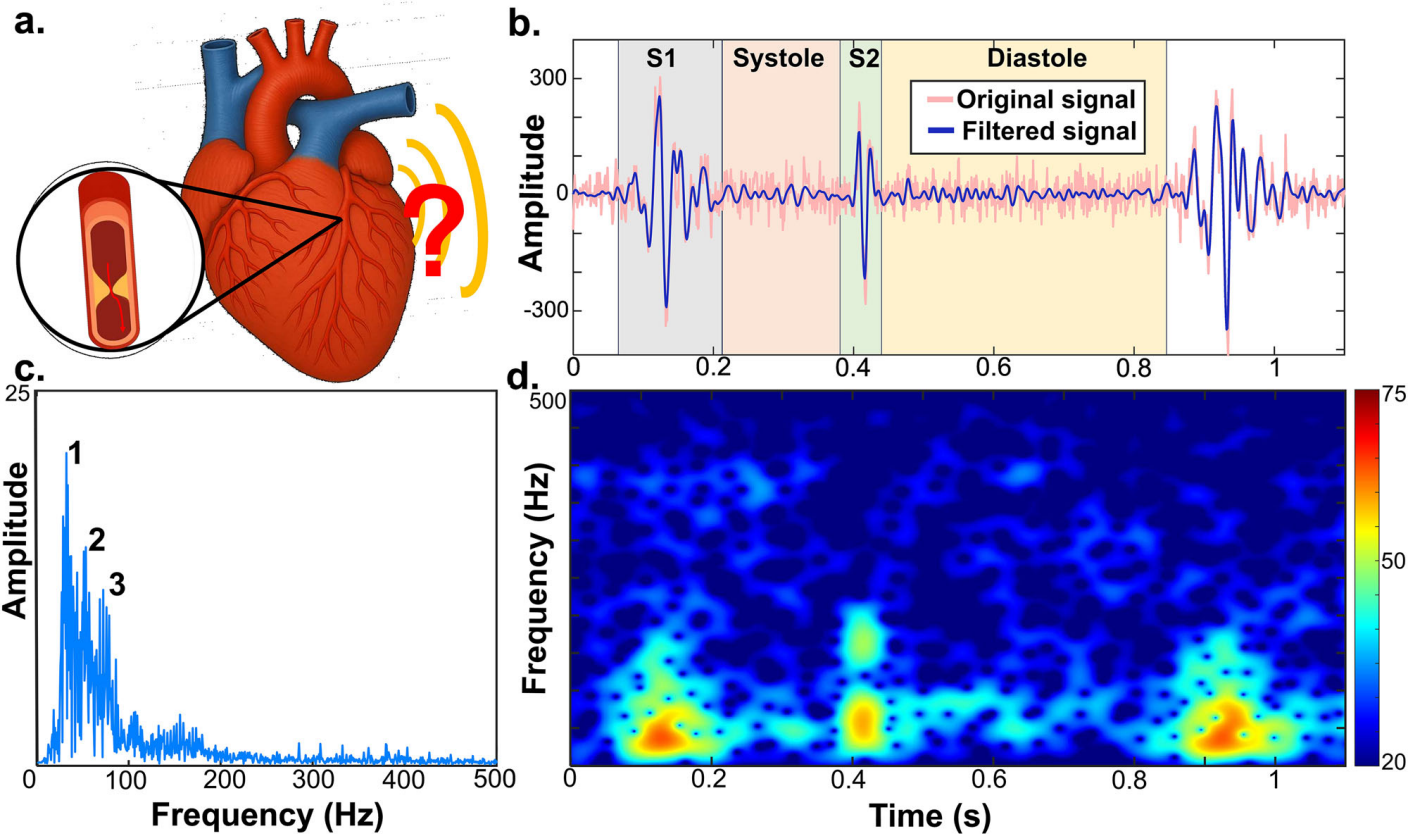
Heart sound signal, frequency spectrum, and time–frequency representation in CAD. **a** Coronary artery stenosis is assumed to induce murmurs near the narrowed vessel. **b** Example of raw (pink) and filtered (blue) heart sound signals from a patient with CAD. **c** Fast Fourier transform of heart sounds within 0–500 Hz showing three dominant spectral peaks. **d** Short-time Fourier transform illustrating temporal evolution of acoustic energy across frequencies.

Figure 2 illustrates signal processing methods commonly used to extract acoustic features for CAD diagnosis^12,16,17^. In the time domain, features such as duration, amplitude, mean, standard deviation, skewness, and kurtosis of S1 and S2 were extracted (Fig. 2b)^18^. In the frequency domain, many studies analyzed the diastolic phase within ~100–500 Hz (Fig. 2c). Akay et al. reported that energy in this range decreases after percutaneous coronary intervention^19^, suggesting that revascularization reduces the turbulent flow responsible for these acoustic components. A further observation from the diastolic spectral analysis is the presence of distinct second and third dominant peaks, which were reported to be more prominent in patients with CAD than in controls^20^. These spectral features became more apparent when high-resolution parametric models such as autoregressive and autoregressive moving average models were applied^21^. Time–frequency representations were also frequently used to derive features, particularly when these were intended as inputs to ML classification or pattern-recognition algorithms (Fig. 2d)^22,23^.

### Frequency analysis for CAD diagnosis

Table 2 summarizes publication year, sample size, frequency threshold, sensitivity, specificity, and accuracy for the 21 signal processing studies. Two consistent patterns can be observed. First, sample sizes increased notably over time, from 24 participants in the earliest study in 1983 to more than 2000 in recent investigations. Second, while reported sensitivity remained relatively stable around 80% across four decades, specificity tended to decline in later studies, which contributed to overall accuracy falling below 70% in larger and more recent cohorts.

**Table 2:** Overview of signal processing studies^a^

Author (year)	Sample size (% CAD)	Frequency thresholds (Hz)	Sensitivity (%)	Specificity (%)	Accuracy (%)	Patient-level separation
Semmlow^12^ (1983)	24 (50)	90	92	92	92	No
Akay^25^ (1991)	31 (71)	400	90	80	80	No
Akay^31^ (1992)	20 (100)	400	85	80	83	Yes
Akay^16^ (1992)	43 (77)	400	93	90	90	No
Akay^20^ (1993)	35 (54)	180	84	81	83	No
Akay^21^ (1993)	80 (60)	300	FFT: 75 AR: 72 ARMA: 71 Eigenvector: 79	63 88 88 91	70 79 78 84	No
Akay^57^ (1995)	100 (64)	300	78	89	82	No
Tateishi^42^ (2001)	168 (76)	700	71	65	66	No
Akay^58^ (2009)	40 (75)	130	77	80	78	No
Makaryus^60^ (2013)	161 (12)	400	90	58	62	No
*Schmidt*^26^ *(2015)*	*133 (47)*	*N/A*	*72*	*65*	*68*	*Yes*
*Azimpour*^61^ *(2016)*	*123 (52)*	*N/A*	*76*	*72*	*74*	*No*
*Winther*^29^ *(2016)*	*255 (25)*	*150*	*76*	*59*	*63*	*Yes*
*Winther*^28^ *(2017)*	*1437 (51)*	*150*	*81*	*53*	*56*	*Yes*
*Schmidt*^27^ *(2019)*	*2033 (10)*	*150*	*89*	*42*	*46*	*Yes*
*Winther*^32^ *(2021)*	*1464 (22)*	*150*	*68*	*57*	*61*	*Yes*
*Renker*^63^ *(2021)*	*226 (82)*	*N/A*	*98*	*15*	*47*	*Yes*
*Lehmacher*^13^ (2022)	*75 (10)*	*N/A*	*88*	*13*	*21*	*Yes*
*Larsen*^41^ *(2022)*	*2222 (12)*	*N/A*	*85*	*49*	*53*	*Yes*
*Schmidt*^65^ *(2022)*	*218 (63)*	*90*	*71*	*64*	*68*	*Yes*
*Rasmussen*^30^ *(2023)*	*1684 (45)*	*100*	*85*	*40*	*61*	*Yes*

*AR* autoregressive, *ARMA* autoregressive moving average, *CAD* coronary artery disease, *FFT* fast Fourier transform.

^a^Italic indicates the studies after the year 2013. All studies focused on the diastolic heart sounds except those by Makaryus, Azimpour, Larsen, and Lehmacher.

**Fig. 3:**
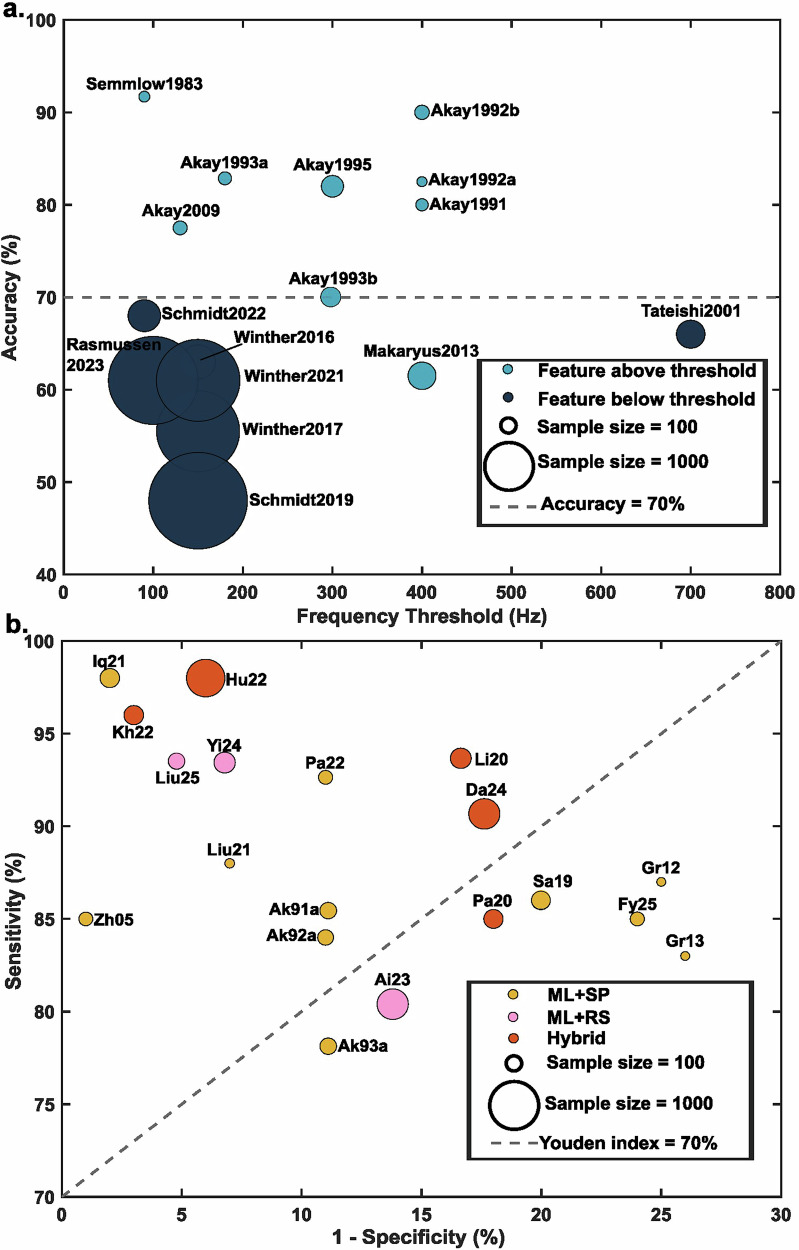
Diagnostic performance of signal processing and machine learning approaches for coronary artery disease detection. Dot size reflects study sample size. **a** Accuracy of signal processing approaches (*n* = 16) across different frequency thresholds. The *y*-axis represents diagnostic accuracy, and the *x*-axis denotes the frequency threshold used. Light blue circles represent studies utilizing features above the specific frequency threshold, while dark blue circles represent features below the threshold. The horizontal dashed line indicates a reference accuracy of 70%. **b** Diagnostic performance of machine learning approaches (*n* = 19). The plot displays Sensitivity (*y*-axis) versus 1−Specificity (*x*-axis). Studies are categorized by methodology: machine learning with signal processing features (ML + SP), machine learning with raw signals (ML + RS), and hybrid models. The diagonal dashed line represents a constant Youden index of 70%.

Most studies (16/21) applied a frequency threshold to quantify diastolic heart-sound energy and compared these values between participants with CAD and controls^24^. Prior to 2013, studies generally focused on frequency components above 150 Hz. For example, Semmlow et al.^12^ and Akay et al.^25^ examined energy above 400 Hz (Fig. 3a) and reported accuracies above 70%. After 2013, Schmidt et al. identified discriminative components below 150 Hz and tested these features in larger cohorts^22,26,27^ where reported accuracies were below 70%. Similar approaches were adopted in six studies from Winther et al.^28,29^, Schmid et al.^27^ and Rasmussen et al.^30^, and as sample sizes increased from 218 to 2033, reported accuracies decreased from 68% to 46%. These trends suggest that high-frequency features may yield higher accuracy in small cohorts, whereas low-frequency features may perform less effectively in larger datasets.

Furthermore, differences in reported diagnostic performance between early studies by Akay and colleagues^16,20,21,25,31^ and later studies by Schmidt and colleagues^27–29,32^ on frequency analysis are likely influenced by variation in recording technology and evaluation procedures. The early studies by Akay were conducted when hardware for capturing subtle intracoronary acoustic signatures was less mature, and some of the recorded murmurs might have reflected secondary hemodynamic conditions rather than primary coronary stenosis. In contrast, Schmidt and colleagues pre-specified low-frequency acoustic features and evaluated them on independent datasets with patient-level separation, a design that may yield lower but more reliable accuracy estimates (Table 2). It is notable that early smaller-cohort studies were predominantly conducted by Akay’s group, whereas later larger-cohort studies were mainly from Winther’s group, suggesting that observed performance differences may partly reflect research group-specific methodologies.

### Machine learning models

ML for CAD diagnosis was first reported in 1991 by Akay et al.^33^. Across the literature, the ML approaches can be grouped into three general families, as summarized in the “Methods type” column in Table 3. The first family (*n* = 11) used multiple handcrafted heart-sound features derived through signal processing and input these features into conventional classifiers (ML + SP). Early studies conducted between 1991 and 2005 were typically single-center, used only one type of microphone, and relied on relatively shallow models such as SVM. Even under these conditions, several studies reported accuracies above 80%^18,34,35^, which suggests that clinically informative patterns may already be present in handcrafted acoustic features. The second family uses either the raw heart sound signal or a time–frequency spectrogram as input to convolutional architectures (ML + RS). This direction has emerged only recently and is represented by three studies published since 2023. Its current application remains limited, possibly because the resulting models tend to have higher architectural complexity and lower interpretability, which could make their integration into clinical workflows more challenging at the current stage. The third family integrates the two strategies described above in a multi-stage or unified pipeline (hybrid). In these studies, ML models, often CNN-based, first extract or select features from the raw signal, and the learned representations are then combined with signal-processing-derived multiple features before classification using either the same model or another model, such as SVM, a convolutional network, or a VGG network. Since around 2020, research has increasingly adopted this hybrid approach. Architectures such as VGG and multiscale attention CNN have been used to unify feature extraction and classification within a single process, which may reduce reliance on hand-crafted features^36^. More recent work has further expanded the feature space to include high-dimensional and multi-domain acoustic characteristics, in some cases surpassing 300 features^14,36^. This trend indicates an ongoing effort to incorporate richer acoustic information while maintaining model robustness and interpretability.

**Table 3:** Overview of machine learning studies

Author (year)	Classifier	Methods type	Sample size (CAD%)	Train:Validation:Test	Patient-level separation	Window	Sen (%)	Spe (%)	Acc (%)	Feature extraction model	Heart sound features
Akay^33^ (1991)	FMMNN	ML + SP	112 (57)	15:0:41	Yes	Diastole	85	89	87	No	Diastolic heart sounds were transformed using a fast wavelet transform, and coefficient-extrema statistics (mean, variance, skewness, and kurtosis) from the third wavelet band were extracted.
Akay^39^ (1992)	MLP	ML + SP	100 (63)	3:0:22	Yes	Diastole	84	89	86	No	Three power peaks and poles from the autoregressive method
Akay^34^ (1993)	MLP	ML + SP	112 (63)	3:0:25	Yes	Diastole	78	89	82	No	Estimated autocorrelation functions and prediction coefficients
Zhao^18^ (2005)	SVM	ML + SP	77 (52)	40:0:37	No	Diastole	85	100	92	No	Mean, SD, skewness, and kurtosis of weighted instantaneous frequency
Griffel^49^ (2012)	SVM	ML + SP	31 (48)	NA	Unclear	Diastole	87	75	81	No	Automutual information function
Griffel^59^ (2013)	SVM	ML + SP	31 (48)	16:0:15	Yes	Diastole	83	74	81	No	Entropy-based features
Samanta^52^ (2019)	SVM	ML + SP	145 (48)	4:0:1	Yes	Full	86	80	83	No	Energy, entropy, variance, skewness, kurtosis from WPD nodes
Li^47^ (2020)	MLP	Hybrid	175 (69)	4:0:1	Yes	Full	94	83	90	CNN	Time, frequency, entropy, and ML features from MFCC images
Pathak^62^ (2020)	KNN	ML + SP	150 (50)	4:0:1	Yes	Full	85	82	85	No	Time–frequency features
Iqtidar^40^ (2021)	MSVM	ML + SP	153 (51)	9:0:1	No	Full	98	98	98	No	Time, frequency, and entropy domain features
Liu^15^ (2021)	SVM	ML + SP	36 (58)	4:0:1	Yes	N/A	88	93	91	No	Time, frequency, and entropy domain features
Pathak^36^ (2022)	MKL	Hybrid	80 (50)	3:1:1	Yes	Full	92	88	90	VGG	Entropy and signals
Khan^64^ (2022)	SVM	Hybrid	155 (52)	7:1:2	Unclear	Full	96	97	96	CNN	MFCC, LPC, RMS, zero-crossing rate
Huang^38^ (2022)	RF	Hybrid	605 (34)	14:3:3	Yes	Full	98	94	96	CNN	MFCC, time-domain, and time–frequency features
Ainiwaer^14^ (2023)	VGG	ML + RS	400 (54)	29:3:8	Yes	Full	80	86	83	VGG	Only signals
Dai^37^ (2024)	CNN	Hybrid	396 (54)	237:79:80	Yes	Full	91	82	88	CNN	Multi-domain features
Yin^66^ (2024)	MACCN	ML + RS	184 (55)	117:30:37	Yes	Full	93	93	93	MACNN	Only signals
Liu^67^ (2025)	TripletNet-HL	ML + RS	110 (54)	3:1:1	Yes	Full	93	95	94	CNN	Only signals
Fynn^68^ (2025)	SVM	ML + SP	80 (50)	4:0:1	Yes	Full	85	76	80	No	Energy, envelope shape, entropy, band power in 20–800 Hz range

*Acc* accuracy, *CAD* coronary artery disease, *CNN* convolutional neural network, *FMMNN* fuzzy min–max neural networks, *KNN* *K*-nearest neighbors, *LR* logistic regression, *MACNN* multiscale attention CNN, *MSVM* multi-support vector machines, *MFCC* Mel frequency cepstral coefficients, *ML* machine learning, *MLP* multilayer perceptron, MKL multiple kernel learning, *NN* neural networks, *RF* random forests, *SD* standard deviation, *Sen* sensitivity, *Spe* specificity, *SVM* support vector machine, *VGG* visual geometry group, *HL* hybrid loss, *ML* machine learning, *SP* signal processing, *RS* raw signal.

### Performance of machine learning for CAD diagnosis

Figure 3b summarizes the diagnostic performance of ML approaches. Among the three methodological families, ML + SP shows the greatest performance variability, with reported specificity ranging from 75% to 99% and sensitivity from 78% to 98%, depending on the specific feature set, classifier choice, and dataset characteristics (sample sizes 31–153). ML + RS demonstrates more consistent specificity, generally around 85–95%, with less variation across studies and sample sizes between 110 and 400. The hybrid approach tends to achieve higher sensitivity, ~85–87%, in studies with sample sizes ranging from 80 to 605.

Model performance also appeared to depend on dataset size. SVM often performed well in smaller datasets with fewer than 200 cases. For example, Liu et al.^15^ (*n* = 36) and Zhao et al.^18^ (*n* = 77) both reported accuracies above 90% when using SVM. This may reflect the margin-maximization principle of SVM, which can help mitigate overfitting in limited sample settings. As datasets increased in size, more complex nonlinear models such as deep learning architectures showed improved performance. For instance, Ainiwaer et al.^14^ (*n* = 400), Dai et al.^37^ (*n* = 396) and Huang et al.^38^ (*n* = 605) all reported high diagnostic accuracy using deep learning methods.

The division of training and validation data also influenced model performance. Some early studies trained models on relatively small datasets while evaluating them on larger test sets^39^, which might have contributed to overestimated performance such as the specificity of 100% reported by Zhao et al.^18^. More recent studies generally used more balanced data partitioning, allocating the largest portion of data to model training, a smaller portion to testing, and a separate portion for validation and parameter tuning. The multi-SVM method proposed by Iqtidar et al.^40^ reported sensitivity, specificity, and accuracy of 98%, although the absence of patient-level separation suggests a possibility of data leakage.

Of note, all included ML studies were conducted at single centers, and in most cases, the test sets included fewer than 50 patients, with approximately half of the cases classified as CAD. Although the reported sensitivity, specificity, and accuracy values are generally high, these results will require confirmation in larger, multicenter prospective studies.

### Machine learning outperforms signal analysis

**Fig. 4:**
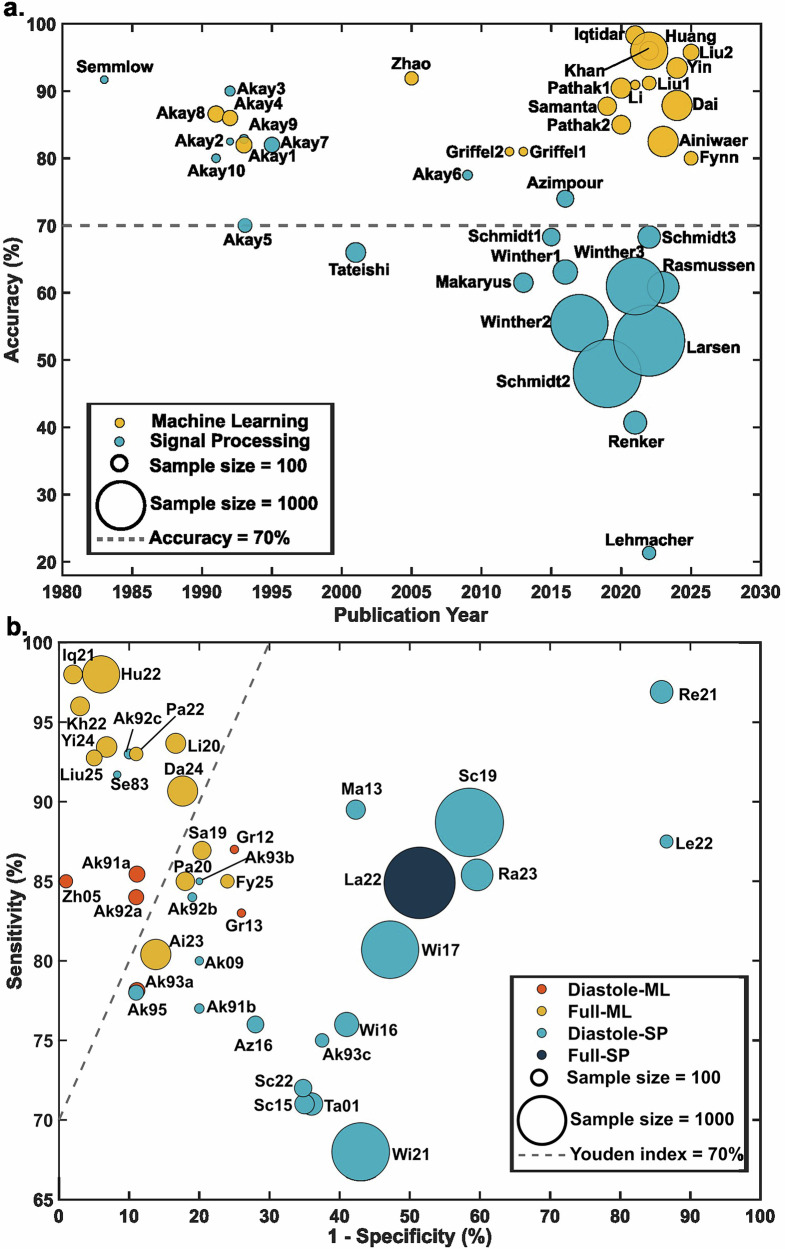
Comparison of diagnostic approaches. Dot size represents sample size. **a** Trends in diagnostic accuracy of acoustic CAD detection methods over time (*n* = 40). The dashed gray line (accuracy = 70%) indicates the separation in reported accuracy between signal processing and machine learning studies. **b** Machine learning and signal processing approaches (*n* = 39) comparing full-cycle and diastolic analyses. ML machine learning, SP signal processing. Dot size reflects sample size, and the dashed reference line marks a Youden index of 70%.

Figure 4a shows that as study sample sizes increased over time, reported diagnostic accuracy in signal processing studies (blue dots, *n* = 21) tended to decline, whereas accuracy in ML studies (yellow dots, *n* = 19) remained relatively stable or showed slight improvement. Since 2000, nearly all signal processing studies have reported accuracy below 70%, while all ML studies have reported accuracy above 80%.

Variation across signal processing studies was also considerable, both between and within research groups. For example, Semmlow et al.^12^ and Akay et al.^21^ from the same group reported accuracies between 70% and 92%, while studies from Schmidt et al.^26^, Winther et al.^28^, and Huang et al.^38^ analyzing larger cohorts reported lower accuracies ranging from 48% to 68%. These differences likely reflect variations in filtering strategies, feature extraction approaches, and evaluation design. In contrast, reported results from ML studies showed greater consistency. Akay et al. conducted three studies with relatively small cohorts (*n* < 150)^33,34,39^ and reported accuracies above 80%, and several subsequent studies from other groups have also achieved comparable performance despite differences in data collection and validation protocols.

Among the included studies, patient-level data separation was adopted in 15 of 19 ML studies (Table 3), compared with 11 of 21 signal processing studies (Table 2). Supplementary Fig. S1 summarizes the performance of studies using patient-level separation. ML studies remained clustered in the high-performance region, with sensitivities of 78–98% and specificities of 74–94%. In contrast, SP studies showed lower and more heterogeneous performance, with sensitivities of 68–93% and specificities of 13–80%. Consistently, Tables 2 and 3 indicate that accuracies in ML studies ranged from 80% to 96%, whereas SP studies reported accuracies ranging from 21% to 83%. Within the methodological constraints assessed in this review, the higher performance reported in ML studies does not appear to be fully explained by readily identifiable methodological issues (e.g., lack of patient-level separation).

### Full-cycle versus diastolic heart sounds for CAD classification

Figure 4b shows the relationship between sensitivity and 1−specificity across studies and distinguishes between signal processing and ML approaches, as well as between analyses using full-cycle and diastolic heart sound data. The gray dashed line indicates a Youden index of 0.7, which provides a reference point for potential clinical applicability. Most studies above this line used ML methods, and those that used full-cycle heart sound inputs generally reported higher discrimination, whereas studies limited to diastolic inputs tended to show lower sensitivity, often around 85%.

In comparison, signal processing studies showed less variation between full-cycle and diastolic analyses. The single full-cycle signal processing study (La22) reported a sensitivity of 85% but a lower specificity of 50%^41^, which aligns with the broader tendency of signal processing results to cluster around the diastolic-based analyses.

These observations suggest that ML models may benefit from information distributed across the entire cardiac cycle, while signal processing methods typically rely on predefined segmentation of S1 and S2 and handcrafted feature extraction. As a result, the performance of signal processing approaches appears less influenced by whether full-cycle or diastolic heart sound data are used.

## Discussion

This systematic review indicates that, within the available evidence, studies using ML approaches have generally reported higher diagnostic performance than conventional signal-processing approaches for heart sound-based CAD diagnosis, although this cross-study pattern should be regarded as a descriptive synthesis and interpreted with caution due to dataset and methodological heterogeneity. Over time, research in this field has progressed from small-scale investigations employing handcrafted acoustic features and standard classifiers to larger studies adopting multi-domain inputs and hybrid deep learning models. These more recent models tend to perform best when using acoustic information from the full cardiac cycle rather than restricting analysis to the diastolic phase, which suggests that diagnostically informative features may be distributed throughout the heart-sound signal rather than confined to diastole alone.

The potential clinical relevance of these findings lies in the possibility that heart sound analysis, as a noninvasive and relatively low-cost method, could complement existing diagnostic pathways for CAD. Possible applications include early triage of patients presenting with chest pain at first medical contact in hospital settings, and remote monitoring if integrated into wearable devices or smartphone applications^42^. However, the translation of these methods into routine practice will require further evidence regarding model robustness, reproducibility in diverse populations, and integration into clinical workflows. This review further extends prior work by jointly examining ML and signal processing approaches^8,43^ and by summarizing how methodological factors, including feature extraction strategies, dataset size, acoustic acquisition protocols, and data partitioning procedures, may contribute to variability in reported diagnostic performance.

The apparent performance advantage of ML over signal processing approaches is likely related to its capacity for end-to-end learning, automatic feature representation, multi-dimensional feature integration, and recognition of complex acoustic patterns. These capabilities can be seen in the studies by Ainiwaer et al.^14^, Dai et al.^37^, and Huang et al.^38^. Ainiwaer et al. used raw time-domain heart-sound signals as input for end-to-end learning. Dai et al. incorporated additional frequency-domain and entropy features using the same dataset. Huang et al. further extended this line of development by introducing spectrogram representations to capture temporal variations in frequency content. Reported diagnostic accuracy improved from 83% in Ainiwaer et al. to 88% in Dai et al. and to 96% in Huang et al., accompanied by a shift from one-dimensional models with different input feature sets^14,37^ to a two-dimensional spectrogram-based architecture^38^. This transition appears to allow richer feature representation, which may have contributed to the observed performance gains.

A recent systematic review of deep learning for heart sound analysis also notes that convolutional and recurrent neural network-based models are predominant^35^. However, that review emphasizes that heterogeneity in evaluation protocols and datasets makes direct cross-study comparison challenging. Many of the high accuracies reported in the literature are based on validation cohorts with fewer than 50 patients, which raises concerns regarding overfitting and limits conclusions about generalizability. Thus, the overall findings that ML usually outperforms conventional signal processing in CAD diagnosis should be interpreted with caution, particularly given variability across study populations, preprocessing pipelines, and model configurations. Studies that enforced patient-level separation between training and testing datasets generally reported lower accuracy, reflecting reduced performance on previously unseen patients. Addressing this issue remains important for future work. In addition, the intrinsic complexity of ML models and the limited interpretability of their decision processes may reduce clinical confidence and constrain practical deployment. Efforts to improve model transparency and provide clinically meaningful explanations will likely be necessary to support real-world adoption.

Beyond the choice of ML model, the temporal selection of the heart sound signal for analysis also influences CAD classification performance. Using the entire cardiac cycle as input generally results in higher accuracy than using only the diastolic segment. This observation suggests that ML models may utilize acoustic patterns distributed across the whole signal rather than relying solely on coronary flow-related murmurs that are expected to occur during diastole. In addition, CAD is known to affect myocardial contractility and hemodynamics, which can alter the intensity, frequency, and duration of S1 and S2^44–46^. It is therefore possible that ML models capture these additional signal changes when trained on full-cycle recordings. Findings from several studies support this interpretation. For example, models that incorporated extra rhythm-related features such as cardiac cycle duration and diastolic duration achieved classification accuracies exceeding 88%, 90%, and 91%^15,37,47^. These results indicate that features outside the diastolic interval may contribute meaningfully to CAD discrimination. In this context, ML may serve not only as a classification tool but also as a means of generating hypotheses about the physiological basis of acoustic markers relevant to CAD. Taken together, these observations suggest that further mechanistic research may benefit from examining heart sound components beyond diastolic coronary murmurs.

Despite its strengths in interpretability and generalizability, signal processing approaches have generally shown modest diagnostic performance for CAD. This may be related to its reliance on predefined restricted features informed by coronary blood flow physiology and its potentially limited ability to capture and integrate nonlinear patterns present in heart sound signals. Physiologically, a stenotic coronary artery may generate flow disturbances that propagate to the chest wall and manifest as murmurs during early diastole. However, the small diameter of epicardial coronary arteries (up to 4 mm), low coronary flow rates (1–1.5 ml/s), the compliance of the arterial walls, and the dampening effects of the lung and chest structures may reduce murmur amplitude below the audible noise floor. As a result, detecting coronary murmurs can be challenging in practice, and conventional signal processing methods may struggle to isolate meaningful features^8,48^. For example, Schmidt et al. found no significant differences in diastolic frequency components above 100 Hz between CAD and non-CAD subjects, suggesting that high-frequency murmurs may not provide reliable discrimination in real-world settings^22^. In subsequent work, the same group shifted their focus to low-frequency diastolic energy, although reported classification accuracy remained below 60%^27^. In addition, conventional spectral approaches may be limited in capturing nonlinear temporal characteristics of heart sounds. Griffel et al. demonstrated such nonlinear patterns by identifying the lag at which the automutual information function decayed to 1/*e* of its initial value in diastolic segments^49^. The use of patient-level data separation also affects performance outcomes. When separation is properly implemented, reported accuracy often decreases, in some cases dropping below 50%, reflecting reduced data leakage and increased exposure to variability across centers, devices, and recording environments. These results suggest that heart sound signals may be too complex to be reliably represented using only a small number of handcrafted features alone. Although methodological factors account for most of the observed variability, the generally consistent microphone quality and recording setups across studies make it difficult to assess the specific impact of hardware on performance. Nevertheless, signal processing remains an active area of investigation due to advantages such as high interpretability, low computational requirements, and minimal parameter tuning, which are valuable for clinical integration and transparent decision-making.

Future research on heart sound-based CAD diagnosis may benefit from several considerations. To enhance clinical applicability, improving model interpretability will be important. Explainable ML techniques such as attention-based visualization may help clarify the basis of model decisions and thereby support clinician trust^50^. For example, Poterucha et al. have shown that EchoNext performs well in detecting structural heart disease^51^, and similar interpretability strategies may be transferable to CAD classification using heart sounds. Current systematic review also indicates that lightweight ML models such as SVM can achieve diagnostic performance comparable to that of more complex hybrid deep learning models, including multiple kernel learning^52^. Model selection should therefore balance dataset size, algorithmic complexity, and interpretability^53^. For smaller datasets (e.g., *n* < 200), SVMs may be more appropriate because they require fewer samples and facilitate clearer model interpretation. In contrast, for larger datasets (*n* > 200), deep learning models can offer advantages in accuracy and efficiency by automatically learning feature representations. Among deep learning approaches, convolutional recurrent neural networks may be particularly suitable for heart sound analysis because they can capture both spectral patterns and temporal dynamics, and they can integrate multi-dimensional representations using time-frequency inputs or signal processing-derived features^54^. For feature selection, in addition to conventional frequency-domain characteristics, future studies could examine time-frequency and nonlinear dynamic features that reflect cardiac functional changes, such as diastolic frequency variations or irregular heart cycle patterns^8^. From a clinical perspective, early identification of CAD often prioritizes sensitivity, and many current ML systems reflect this need. However, improving specificity without sacrificing sensitivity will be essential for real-world applications. Most included studies were single-center; thus, external validation across multiple centers and diverse patient populations is necessary to establish generalizability. Also, consistent documentation of recording environments and microphone characteristics is needed to clarify how acquisition conditions influence performance and to support reproducibility. Moreover, it will also be important to exclude patients with confounding secondary conditions, such as mitral regurgitation, using echocardiography before the heart sound measurement. Finally, future deployment may include integration of ML models into smartphone-based applications such as FonoCheck, which could enable large-scale, home-based population screening using widely accessible devices^55,56^. Achieving this goal will require systematic evaluation in clinical and community settings to ensure feasibility, reliability, and equitable access.

This review has several limitations. First, although 40 studies including 13,814 participants were analyzed, many of the individual studies relied on small single-center datasets, which increases the risk of selection bias and may limit the extent to which the findings represent broader clinical populations. Second, the review focused exclusively on unimodal heart sound-based approaches to ensure methodological comparability across studies. This choice necessarily excluded multimodal strategies that integrate heart sounds with electrocardiogram or imaging data, which may provide additional diagnostic information. Third, only English-language publications were included, which may have resulted in the omission of relevant work and could underrepresent research conducted in regions with a high burden of CAD.

Over the past four decades, heart sound analysis for noninvasive CAD diagnosis has evolved from basic time-domain feature extraction toward more advanced hybrid ML approaches. Although study designs, datasets, and analytical pipelines varied, ML-based methods have generally shown more favorable diagnostic performance than conventional signal-processing techniques. Notably, studies without explicit patient-level separation may report slightly inflated performance due to potential data leakage. Nonetheless, further research is required to enhance interpretability, assess model performance in broader clinical settings, and address practical considerations for large-scale deployment. Progress in these areas will be important for supporting the eventual integration of heart sound-based CAD assessment into routine clinical workflows.

## Methods

### Search strategy

A systematic literature search was conducted on 1 June 2025 in PubMed, Web of Science, Embase, and the Cochrane Library using the following search terms: “(“coronary stenosis”[MeSH Terms] OR “coronary artery stenosis”[Title/Abstract] OR “coronary narrowing”[Title/Abstract] OR “coronary disease”[MeSH Terms] OR “coronary artery disease*“[Title/Abstract]) AND (“heart sound*“[Title/Abstract] OR “murmur*“[Title/Abstract] OR “phonocardiogr*“[Title/Abstract] OR “cardiac sound*“[Title/Abstract]).” All retrieved records were imported into Zotero (7.0.15) for removal of duplicates and further processing.

### Inclusion and exclusion criteria

Studies were included if they focused on heart-sound analysis for CAD diagnosis in humans and confirmed CAD using coronary angiography or coronary computed tomography angiography. In our study, CAD was defined as ≥50% luminal diameter stenosis in any main coronary artery, including the left main, left anterior descending, left circumflex, or right coronary artery^4^.

A study was categorized into the ML group if it employed machine learning or deep learning algorithms, such as support vector machines (SVMs) or convolutional neural networks (CNNs) for CAD classification. To facilitate comparison with ML approaches, studies that primarily relied on hand-crafted signal-processing features and used rule-based scoring or classical parametric discriminant analysis, such as linear discriminant analysis or quadratic discriminant analysis, were grouped as signal-processing methods. The primary outcomes of interest were diagnostic performance metrics, including accuracy, sensitivity, and specificity. Only peer-reviewed, full-text original research articles were considered. We excluded abstract-only publications, letters, editorials, commentaries, non-peer-reviewed papers, retracted articles, reviews, and case reports. Animal studies and studies that did not report a CAD diagnostic method were also excluded. In addition, studies were excluded if they did not clearly describe their acoustic analysis pipeline or combined heart sound recordings with other diagnostic modalities such as electrocardiography or imaging. Only English-language publications were included, with no geographical restrictions.

### Literature screening

All articles were screened independently by two reviewers (A.A. and H.L.) based on titles and abstracts. Disagreements were resolved through discussion. Full-text screening was then carried out by the same reviewers using the predefined inclusion and exclusion criteria, and any remaining discrepancies were resolved by consensus. Reference lists of eligible studies were also reviewed to identify additional relevant publications.

### Data extraction

The following data were extracted from each eligible study and entered into a predefined table:

- Publication details, including study identifier, author, year, title, and abstract.
- CAD diagnostic method (coronary angiography or coronary computed tomography angiography).
- CAD definition and severity information, such as at least 50% stenosis, as well as whether multiple arteries are involved.
- Heart sound acquisition, including device type (e.g., electronic stethoscope or wearable sensor) and recording protocol (e.g., duration and noise reduction procedures).
- Analytical pipeline including signal processing techniques (e.g., wavelet transformation or spectral analysis), feature definitions (e.g., S1/S2 amplitude ratio or entropy measures), and ML algorithms (e.g., CNNs for classification or SVMs for feature selection).
- Validation strategy, including whether patient-level data separation was implemented (Yes/No/Unclear).
- Diagnostic performance, including accuracy, sensitivity, and specificity.

### Statistical analysis

Due to substantial heterogeneity in study populations, recording hardware, and analytical pipelines, a formal meta-analysis was not performed. Instead, descriptive statistics and exploratory data visualizations were used to summarize the findings. Continuous variables such as sample size were reported as medians, and categorical variables as counts and percentages. Each bubble plot represented one study, with the bubble area proportional to sample size. The plots illustrated (i) the relation between frequency thresholds and reported accuracy in signal processing studies, (ii) sensitivity versus 1−specificity for studies using ML approaches, (iii) reported accuracy by publication year for signal processing and ML studies, and (iv) sensitivity versus 1-specificity comparing analyses using the full cardiac cycle and the diastolic phase only, with a reference line corresponding to a Youden index of 0.7.

When accuracy was not reported, it was calculated manually from the published data using Eq. (1):

1 $$Accuracy=\frac{TP+TN}{TP+FP+FN+TN}\,$$

where TP, FP, FN, and TN denote true positives, false positives, false negatives, and true negatives, respectively. All statistical analyses were performed in Matlab R2024a.

## Supplementary information

Supplementary.Material21.01.

## Data Availability

All data analyzed in this study were obtained from published literature and are fully presented in the tables and figures within the manuscript.
