# Supplementary material for: Coronary artery disease diagnosis with signal processing and machine learning of heart sound signals: a systematic review
Source: NPJ Digit Med. 2026 Mar 16;9:350. doi: 10.1038/s41746-026-02530-8 (PMC13136408; doi:10.1038/s41746-026-02530-8)
Supplement: Supplementary file 1 — Supplementary.Material21.01. [file 41746_2026_2530_MOESM1_ESM.pdf]

SUPPLEMENTAL MATERIAL

Coronary artery disease diagnosis with signal processing and machine learning of heart sound signals: a systematic review

Aikeliyaer Ainiwaer[1], Tom J.A.J. Konings[2], Kaisaierjiang Kadier[3], Xiang Ma[3], Muhammet Emin Akpulat[1], Frits W. Prinzen[1], Tammo Delhaas[2], Hongxing Luo[1*].

1. Department of Physiology, Cardiovascular Research Institute Maastricht (CARIM), Maastricht University, 6229 ER Maastricht, the Netherlands.

2. Department of Biomedical Engineering, Cardiovascular Research Institute Maastricht (CARIM), Maastricht University, 6229 ER Maastricht, the Netherlands.

3. Department of Cardiology, First Affiliated Hospital of Xinjiang Medical University, Urumqi, Xinjiang, 830000, China.

* **Correspondence to:**

Hongxing Luo, MD, PhD, Department of Physiology, Cardiovascular Research Institute Maastricht, Maastricht University, P.O. Box 616, 6200 MD Maastricht, the Netherlands. Email: h.luo@maastrichtuniversity.nl. Telephone: +31 (0)685704703.

SUPPLEMENTAL MATERIAL

Table of Contents

Table S1. Diagnostic details for included studies.. 3

Table S2. Completed Preferred Reporting Items for Systematic Reviews and Meta-analyses (PRISMA) 2020 statement..5

Figure S1. Machine learning and signal processing approaches used for CAD diagnosis, categorized based on whether patient-level separation was implemented..10

References..10

SUPPLEMENTAL MATERIAL

Table S1. Diagnostic details for included studies (n=40).

Author (year)	Methods	CAD definition	≥50% (n%)	≥70% (n%)	Single vessel ≥50% (%)	Multiple vessels ≥50% (%)
Semmlow[1] (1983)	CAG	≥50%	7 (29%)	5 (21%)	6 (25%)	1 (4.2%)
Akay[2] (1991)	CAG	≥50%	20 (64%)	20 (64%)	16 (52%)	4 (13%)
Akay[3] (1991)	CAG	Not clear				
Akay[4] (1992)	CAG	≥50%	20(100%)	18(90%)		
Akay[5] (1992)	CAG	≥50%	27(77%)	25(71%)	14(40%)	13(37%)
Akay[6] (1992)	CAG	Not clear				
Akay[7] (1993)	CAG	≥50%	15(43%)	12(34%)	10(29%)	5(14%)
Akay[8] (1993)	CAG	Not clear				
Akay[9] (1993)	CAG	≥50%	48(60%)	37(46%)	32(40%)	8(10%)
Akay[10] (1995)	CAG	≥25%	60(60%)	43(43%)	32(32%)	28(28%)
Tateishi[11] (2001)	CAG	≥50%	84 (50%)	51(30%)		
Zhao[12] (2005)	CAG	Not clear				
Akay[13] (2009)	CAG	Not clear				
Griffel[14] (2012)	CAG	≥50%	15(48%)			
Griffel[15] (2013)	CAG	≥50%	15(48%)			
Makaryus[16] (2013)	cCTA	≥50%	19(12%)	5(3%)		
Schmidt[17] (2015)	CAG	≥50%	63(47%)		43(32%)	20(15%)
Azimpour[18] (2016)	CAG	≥50%	64(52%)	54(44%)	22(18%)	41(33%)
Winther[19] (2016)	CAG and cCTA	≥50%	63(28%)	28(12%)	44(19%)	19(8%)
Winther[20] (2017)	CAG	≥50%	153(11%)		94(7%)	59(4%)
Schmidt[21] (2019)	CAG	≥50%	212(9%)			
Samanta[22] (2019)	CAG	≥50%	33(50%)		4(6%)	29(44%_
Li[23] (2020)	CAG	≥50%	120(69%)			
Pathak[24] (2020)	CAG	≥50%	40(50%)		9(11%)	31(39%)
Winther[25] (2021)	CAG	≥50%				
Iqtidar[26] (2021)		Not clear				
Liu[27] (2021)	CAG	≥50%	21(58%)		5(14%)	16(44%)
Renke[28] (2021)	CAG	≥50%	89(39%)	62(29%)	52(23%)	37(42%)
Larsen[29] (2022)	CAG	≥50%	257(12%)			
Pathak[30] (2022)	CAG	≥50%	40(50%)		9(11%)	31(39%)
Khan[31] (2022)		Not clear				
Huang[32] (2022)	CAG	≥50%	206(37%)			
Schmidt[33] (2022)	CAG	≥50%	137(63%)		73(34%)	64(47%)
Lehmacher[34] (2022)	CAG	≥50%r				
Rasmussen[35] (2023)	CAG	≥50%	217(13%)		131(8%)	68(4%)
Ainiwaer[36] (2023)	CAG	≥50%	51(64%)		21(26%)	30(38%)
Dai[37] (2024)	CAG	≥50%	51(64%)			
Yin[38] (2024)	CAG	≥50%				
Liu[39] (2025)	CAG	≥50%	59(54%)			

Fynn[40] (2025)	CAG	≥50%	40(50%)		14(18%)	26(33)

White rows represent studies that explicitly define CAD, whereas gray rows represent studies that do not provide an explicit CAD definition in the article. Empty cells indicate that the corresponding data were not reported in the publications. CAG: Coronary Angiography. cCTA: coronary computed tomography angiography.

SUPPLEMENTAL MATERIAL

Table S2. Completed Preferred Reporting Items for Systematic Reviews and Meta-analyses (PRISMA) 2020 statement.

Section and Topic	Item #	Checklist item	Location where item is reported
**TITLE**			
Title	1	Identify the report as a systematic review.	Page1
**ABSTRACT**			
Abstract	2	See the PRISMA 2020 for Abstracts checklist.	Page2
**INTRODUCTION**			
Rationale	3	Describe the rationale for the review in the context of existing knowledge.	Page3
Objectives	4	Provide an explicit statement of the objective(s) or question(s) the review addresses.	Page3
**METHODS**			
Eligibility criteria	5	Specify the inclusion and exclusion criteria for the review and how studies were grouped for the syntheses.	Page13
Information sources	6	Specify all databases, registers, websites, organisations, reference lists and other sources searched or consulted to identify studies. Specify the date when each source was last searched or consulted.	Page13
Search strategy	7	Present the full search strategies for all databases, registers and websites, including any filters and limits used.	Page13
Selection process	8	Specify the methods used to decide whether a study met the inclusion criteria of the review, including how many reviewers screened each record and each report retrieved, whether they worked independently, and if applicable, details of automation tools used in the process.	Page14
Data collection process	9	Specify the methods used to collect data from reports, including how many reviewers collected data from each report, whether they worked independently, any processes for obtaining or confirming data from study investigators, and if applicable, details of automation tools used in the process.	Page4
Data items	10a	List and define all outcomes for which data were sought. Specify whether all results that were compatible with each outcome domain in each study were sought (e.g. for all measures, time points, analyses), and if not, the methods used to	Page14

Section and Topic	Item #	Checklist item	Location where item is reported
		decide which results to collect.	
	10b	List and define all other variables for which data were sought (e.g. participant and intervention characteristics, funding sources). Describe any assumptions made about any missing or unclear information.	Page14
Study risk of bias assessment	11	Specify the methods used to assess risk of bias in the included studies, including details of the tool(s) used, how many reviewers assessed each study and whether they worked independently, and if applicable, details of automation tools used in the process.	Page14
Effect measures	12	Specify for each outcome the effect measure(s) (e.g. risk ratio, mean difference) used in the synthesis or presentation of results.	NA
Synthesis methods	13a	Describe the processes used to decide which studies were eligible for each synthesis (e.g. tabulating the study intervention characteristics and comparing against the planned groups for each synthesis (item #5)).	Page4
	13b	Describe any methods required to prepare the data for presentation or synthesis, such as handling of missing summary statistics, or data conversions.	Page15
	13c	Describe any methods used to tabulate or visually display results of individual studies and syntheses.	Page15
	13d	Describe any methods used to synthesize results and provide a rationale for the choice(s). If meta-analysis was performed, describe the model(s), method(s) to identify the presence and extent of statistical heterogeneity, and software package(s) used.	Page15
	13e	Describe any methods used to explore possible causes of heterogeneity among study results (e.g. subgroup analysis, meta-regression).	Supplementary Table S2 and S3
	13f	Describe any sensitivity analyses conducted to assess robustness of the synthesized results.	Page7
Reporting bias assessment	14	Describe any methods used to assess risk of bias due to missing results in a synthesis (arising from reporting biases).	Page15
Certainty	15	Describe any methods used to assess certainty (or confidence) in the body of evidence for an outcome.	Page15

Section and Topic	Item #	Checklist item	Location where item is reported
assessment			
**RESULTS**			
Study selection	16a	Describe the results of the search and selection process, from the number of records identified in the search to the number of studies included in the review, ideally using a flow diagram.	Page4, Figure 1
	16b	Cite studies that might appear to meet the inclusion criteria, but which were excluded, and explain why they were excluded.	Page4
Study characteristics	17	Cite each included study and present its characteristics.	Table 1-3, and Supplementary Table S1
Risk of bias in studies	18	Present assessments of risk of bias for each included study.	NA
Results of individual studies	19	For all outcomes, present, for each study: (a) summary statistics for each group (where appropriate) and (b) an effect estimate and its precision (e.g. confidence/credible interval), ideally using structured tables or plots.	Table 2 and 3, Figure 3 and 4
Results of syntheses	20a	For each synthesis, briefly summarise the characteristics and risk of bias among contributing studies.	Table 1-3, and Supplementary Table S1
	20b	Present results of all statistical syntheses conducted. If meta-analysis was done, present for each the summary estimate and its precision (e.g. confidence/credible interval) and measures of statistical heterogeneity. If comparing groups, describe the direction of the effect.	Table 2 and 3
	20c	Present results of all investigations of possible causes of heterogeneity among study results.	Page 6, Supplementary Table S2 and 3

Section and Topic	Item #	Checklist item	Location where item is reported
	20d	Present results of all sensitivity analyses conducted to assess the robustness of the synthesized results.	Table 2 and 3
Reporting biases	21	Present assessments of risk of bias due to missing results (arising from reporting biases) for each synthesis assessed.	NA
Certainty of evidence	22	Present assessments of certainty (or confidence) in the body of evidence for each outcome assessed.	NA
**DISCUSSION**			
Discussion	23a	Provide a general interpretation of the results in the context of other evidence.	Page 9
	23b	Discuss any limitations of the evidence included in the review.	Page 13
	23c	Discuss any limitations of the review processes used.	Page 13
	23d	Discuss implications of the results for practice, policy, and future research.	Page 12
**OTHER INFORMATION**			
Registration and protocol	24a	Provide registration information for the review, including register name and registration number, or state that the review was not registered.	NA
	24b	Indicate where the review protocol can be accessed, or state that a protocol was not prepared.	NA
	24c	Describe and explain any amendments to information provided at registration or in the protocol.	NA
Support	25	Describe sources of financial or non-financial support for the review, and the role of the funders or sponsors in the review.	Page 15
Competing interests	26	Declare any competing interests of review authors.	Page 16
Availability of data, code and other materials	27	Report which of the following are publicly available and where they can be found: template data collection forms; data extracted from included studies; data used for all analyses; analytic code; any other materials used in the review.	Page 15

From: Page MJ, McKenzie JE, Bossuyt PM, Boutron I, Hoffmann TC, Mulrow CD, et al. The PRISMA 2020 statement: an updated guideline for reporting systematic reviews. BMJ 2021;372:n71. doi:

SUPPLEMENTAL MATERIAL

10.1136/bmj.n71. This work is licensed under CC BY 4.0. To view a copy of this license, visit https://creativecommons.org/licenses/by/4.0/

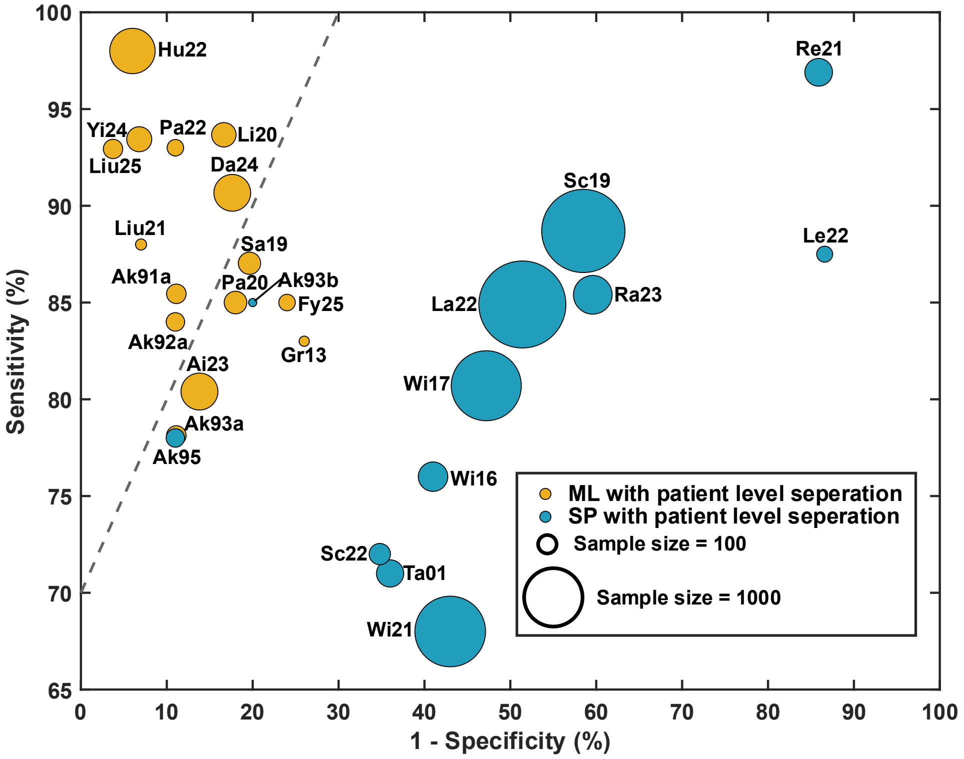

Figure S1. Comparison of diagnostic performance between machine learning and signal processing approaches among studies implementing patient-level separation (n = 26).

Dot size indicates sample size. The dashed grey line (accuracy = 70%) highlights the threshold distinguishing diagnostic performance. ML, machine learning; SP, signal processing.

References

1. Semmlow, J., Welkowitz, W., Kostis, J. & Mackenzie, J. W. Coronary Artery Disease - Correlates Between Diastolic Auditory Characteristics and Coronary Artery Stenoses. *IEEE Trans. Biomed. Eng.* **BME-30**, 136–139 (1983).
2. Akay, M., Welkowitz, W., Semmlow, J. L. & Kostis, J. Application of the ARMA method to acoustic detection of coronary artery disease. *Med. Biol. Eng. Comput.* **29**, 365–372 (1991).
3. Akay, Y. M. Noninvasive Detection of Coronary Artery Disease. in *1991 IEEE International Joint Conference on Neural Networks* 1658–1669 vol.2 (IEEE, Singapore, 1991).
4. Akay, M., Akay, Y. M., Welkowitz, W., Semmlow, J. L. & Kostis, J. B. Application of adaptive filters to noninvasive acoustical detection of coronary occlusions before and after angioplasty. *IEEE Trans. Biomed. Eng.* **39**, 176–184 (1992).
5. Akay, M., Welkowitz, W., Semmlow, J. L., Akay, Y. M. & Kostis, J. Noninvasive acoustical

detection of coronary artery disease using the adaptive line enhancer method. *Med. Biol. Eng. Comput.* **30**, 147–154 (1992).
6. Akay, M. Noninvasive diagnosis of coronary artery disease using a neural network algorithm. *Biol. Cybern.* **67**, 361–367 (1992).
7. Akay, M., Akay, Y. M., Welkowitz, W., Semmlow, J. L. & Kostis, J. Noninvasive characterization of the sound pattern caused by coronary artery stenosis using FTF/FAEST zero tracking filters: Normal/abnormal study. *Ann. Biomed. Eng.* **21**, 175–182 (1993).
8. Akay, M. & Welkowitz, W. Acoustical detection of coronary occlusions using neural networks. *J. Biomed. Eng.* **15**, 469–473 (1993).
9. Akay, Y. M., Akay, M., Welkowitz, W., Semmlow, J. L. & Kostis, J. B. Noninvasive acoustical detection of coronary artery disease: a comparative study of signal processing methods. *IEEE Trans. Biomed. Eng.* **40**, 571–578 (1993).
10. AKAY, M. Harmonic decomposition of diastolic heart sounds associated with coronary-artery disease. *SIGNAL Process.* **41**, 79–90 (1995).
11. Tateishi, O. Clinical significance of the acoustic detection of coronary artery stenosis. *J. Cardiol.* **38**, 255–262 (2001).
12. Zhao Zhidong. Noninvasive Diagnosis of Coronary Artery Disease Based on Instantaneous Frequency of Diastolic Murmurs and SVM. in *2005 IEEE Engineering in Medicine and Biology 27th Annual Conference* 5651–5654 (IEEE, Shanghai, China, 2005).
13. Akay, M. *et al.* Dynamics of Diastolic Sounds Caused by Partially Occluded Coronary Arteries. *IEEE Trans. Biomed. Eng.* **56**, 513–517 (2009).
14. Griffel, B., Zia, M. K., Fridman, V., Saponieri, C. & Semmlow, J. L. Detection of coronary artery disease using automutual information. *Cardiovasc. Eng. Technol.* **3**, 333–344 (2012).
15. Griffel, B., Zia, M. K., Fridman, V., Saponieri, C. & Semmlow, J. L. Path length entropy analysis of diastolic heart sounds. *Comput. Biol. Med.* **43**, 1154–1166 (2013).
16. Makaryus, A. N. *et al.* Utility of an Advanced Digital Electronic Stethoscope in the Diagnosis of Coronary Artery Disease Compared With Coronary Computed Tomographic Angiography. *Am. J. Cardiol.* **111**, 786–792 (2013).
17. Schmidt, S. E., Holst-Hansen, C., Hansen, J., Toft, E. & Struijk, J. J. Acoustic Features for the Identification of Coronary Artery Disease. *IEEE Trans. Biomed. Eng.* **62**, 2611–2619 (2015).
18. Azimpour, F., Caldwell, E., Tawfik, P., Duval, S. & Wilson, R. F. Audible Coronary Artery Stenosis. *Am. J. Med.* **129**, 515-521.e3 (2016).
19. Winther, S. *et al.* Diagnosing coronary artery disease by sound analysis from coronary stenosis induced turbulent blood flow: diagnostic performance in patients with stable angina pectoris. *Int. J. Cardiovasc. Imaging* **32**, 235–245 (2016).
20. Winther, S. *et al.* Diagnostic performance of an acoustic-based system for coronary artery disease risk stratification. *Heart* **104**, 928–935 (2018).
21. Schmidt, S. E. *et al.* Coronary artery disease risk reclassification by a new acoustic-based score. *Int. J. Cardiovasc. Imaging* **35**, 2019–2028 (2019).
22. Samanta, P., Pathak, A., Mandana, K. & Saha, G. Classification of coronary artery diseased and normal subjects using multi-channel phonocardiogram signal. *Biocybern. Biomed. Eng.* **39**, 426–443 (2019).
23. Li, H. *et al.* A fusion framework based on multi-domain features and deep learning features of

phonocardiogram for coronary artery disease detection. *Comput. Biol. Med.* **120**, 103733 (2020).

24. Pathak, A., Samanta, P., Mandana, K. & Saha, G. Detection of coronary artery atherosclerotic disease using novel features from synchrosqueezing transform of phonocardiogram. *Biomed. SIGNAL Process. CONTROL* **62**, (2020).
25. Winther, S. *et al.* Advanced heart sound analysis as a new prognostic marker in stable coronary artery disease. *Eur. Heart J. Digit. Health* **2**, 279–289 (2021).
26. Iqtidar, K., Qamar, U., Aziz, S. & Khan, M. U. Phonocardiogram signal analysis for classification of Coronary Artery Diseases using MFCC and 1D adaptive local ternary patterns. *Comput. Biol. Med.* **138**, 104926 (2021).
27. Liu, T. *et al.* Detection of Coronary Artery Disease Using Multi-Domain Feature Fusion of Multi-Channel Heart Sound Signals. *Entropy* **23**, 642 (2021).
28. Renker, M. *et al.* Prospective validation of an acoustic-based system for the detection of obstructive coronary artery disease in a high-prevalence population. *Heart Vessels* **36**, 1132–1140 (2021).
29. Larsen, B. S. *et al.* Improved pre-test likelihood estimation of coronary artery disease using phonocardiography. *Eur. Heart J. - Digit. Health* **3**, 600–609 (2022).
30. Pathak, A., Mandana, K. & Saha, G. Ensembled Transfer Learning and Multiple Kernel Learning for Phonocardiogram Based Atherosclerotic Coronary Artery Disease Detection. *IEEE J. Biomed. Health Inform.* **26**, 2804–2813 (2022).
31. Khan, M. U. *et al.* A two-stage classification model integrating feature fusion for coronary artery disease detection and classification. *Multimed. Tools Appl.* **81**, 13661–13690 (2022).
32. Huang, Y. *et al.* A customized framework for coronary artery disease detection using phonocardiogram signals. *Biomed. Signal Process. Control* **78**, 103982 (2022).
33. Schmidt, S. E. *et al.* Coronary Artery Disease Detected by Low Frequency Heart Sounds. *Cardiovasc. Eng. Technol.* **13**, 864–871 (2022).
34. Lehmacher, J. *et al.* Diagnostic performance of a device for acoustic heart sound analysis in patients with suspected myocardial infarction. *Open Heart* **10**, (2023).
35. Rasmussen, L. D. *et al.* Likelihood reclassification by an acoustic-based score in suspected coronary artery disease. *Heart Br. Card. Soc.* **109**, 1223–1230 (2023).
36. Ainiwaer, A. *et al.* Deep learning of heart-sound signals for efficient prediction of obstructive coronary artery disease. *Heliyon* **10**, e23354 (2024).
37. Dai, Y. *et al.* Deep learning fusion framework for automated coronary artery disease detection using raw heart sound signals. *Heliyon* **10**, e35631 (2024).
38. Yin, C. *et al.* Detection of Coronary Artery Disease Based on Clinical Phonocardiogram and Multiscale Attention Convolutional Compression Network. *IEEE J. Biomed. Health Inform.* **28**, 1353–1362 (2024).
39. Liu, X., Lv, C., Cao, L. & Guo, X. Detection of coronary artery disease using a triplet network and hybrid loss function on heart sound signal. *Biomed. SIGNAL Process. CONTROL* **104**, (2025).
40. Fynn, M. *et al.* Practicality meets precision: Wearable vest with integrated multi-channel PCG sensors for effective coronary artery disease pre-screening. *Comput. Biol. Med.* **189**, 109904 (2025).
